# In utero exposure to methylphenidate, amphetamines and atomoxetine and offspring neurodevelopmental disorders – a population-based cohort study and meta-analysis

**DOI:** 10.1038/s41380-025-02968-4

**Published:** 2025-03-27

**Authors:** Kathrine Bang Madsen, Henrik Larsson, Charlotte Skoglund, Xiaoqin Liu, Trine Munk-Olsen, Veerle Bergink, Jeffrey H. Newcorn, Samuele Cortese, Paul Lichtenstein, Ralf Kuja-Halkola, Zheng Chang, Brian D’Onofrio, Per Hove Thomsen, Kari Klungsøyr, Isabell Brikell, Miguel Garcia-Argibay

**Affiliations:** 1 Department of Clinical Research, University of Southern Denmark, Odense, Denmark; 2 NCRR- National Centre for Register-based Research, Department of Public Health, Aarhus University, Aarhus, Denmark; 3 CIRRAU - Centre for Integrated Register-based Research, Aarhus University, Aarhus, Denmark; 4 School of Medical Sciences, Faculty of Medicine and Health, Örebro University, Örebro, Sweden; 5 Department of Medical Epidemiology and Biostatistics, Karolinska Institutet, Stockholm, Sweden; 6 Department of Women’s and Children’s Health, Uppsala University, Uppsala, Sweden; 7 Department of Clinical Neuroscience, Karolinska Institutet, Stockholm, Sweden; 8 Department of Psychiatry, Icahn School of Medicine at Mount Sinai, New York, NY USA; 9 Department of Psychiatry, Erasmus Medical Centre Rotterdam, Rotterdam, the Netherlands; 10 Centre for Innovation in Mental Health, School of Psychology, Faculty of Environmental and Life Sciences, University of Southampton, Southampton, UK; 11 Solent NHS Trust, Southampton, UK; 12 Clinical and Experimental Sciences (CNS and Psychiatry), Faculty of Medicine, University of Southampton, Southampton, UK; 13 Hassenfeld Children’s Hospital at NYU Langone, New York University Child Study Center, New York City, NY USA; 14 DiMePRe-J-Department of Precision and Regenerative Medicine-Jonic Area, University of Bari “Aldo Moro”, Bari, Italy; 15 Department of Psychological and Brain Sciences, Indiana University, Bloomington, IN USA; 16 Research Center at the Department for Child‐ and Adolescent Psychiatry, Aarhus University Hospital, Skejby, Denmark; 17 Department of Clinical Medicine, Aarhus University, Aarhus, Denmark; 18 Department of Global Public Health and Primary Care, University of Bergen, Bergen, Norway; 19 Division of Mental and Physical Health, Norwegian Institute of Public Health, Bergen, Norway; 20 Department of Biomedicine, Aarhus University, Aarhus, Denmark

**Keywords:** ADHD, Autism spectrum disorders

## Abstract

The use of Attention-Deficit/Hyperactivity Disorder (ADHD) medications during pregnancy is increasing, raising concerns about potential long-term effects on offspring. This study investigates in utero exposure to methylphenidate, amphetamines and atomoxetine and risk of offspring neurodevelopmental disorders (NDDs). The population-based cohort study identified from Swedish registers included 861,650 children born by 572,731 mothers from 2008–2017. We categorized exposure based on redeemed medication during pregnancy and compared exposed children to those whose mothers discontinued medication before conception. Main outcomes were any NDD, including ADHD and autism spectrum disorder (ASD). Cox proportional hazards regression estimated hazard ratios (HRs), adjusting for maternal psychiatric and sociodemographic factors. Sensitivity analyses included stratifications by medication type, timing, and duration of exposure, and sibling comparisons. We also performed a meta-analysis combining data from the present study with those from a previous Danish study. Results showed no increased risk for any NDD (HR_adjusted_ 0.95, 95% CI 0.82–1.11), ADHD (HR_adjusted_ 0.92, 95% CI 0.78–1.08), or ASD (HR_adjusted_ 0.86, 95% CI 0.63–1.18). Sensitivity analyses showed consistent patterns of no increased risks across different exposure durations, medication types and between siblings. Meta-analyses further supported the findings (pooled HR for any NDD 1.00, 95% CI 0.83;1.20). Our study provides evidence that in utero exposure to ADHD medications does not increase the risk of long-term NDDs in offspring. This study replicates safety data for methylphenidate and extends it with new safety data on amphetamines and atomoxetine. These findings are crucial for informing clinical guidelines and helping healthcare providers and expectant mothers make informed decisions.

## Introduction

Attention-Deficit/Hyperactivity Disorder (ADHD) is the most prevalent neurodevelopmental disorder (NDD), affecting individuals across their lifespan [1]. With the increasing prescription rate of ADHD medications among women of reproductive age [2], there has been a concurrent rise in the use of these medications during pregnancy. Estimates suggest that up to 0.8% of pregnant women in the Nordic countries and over 1% of pregnant women in the United States are currently prescribed ADHD medications, making these among the most commonly used medications during pregnancy [3, 4]. Despite their widespread use, there is still insufficient replicated empirical evidence concerning the long-term safety of in utero exposure to ADHD medications, leading many expectant mothers to discontinue use due to concerns about potential harm to the unborn child [5, 6].

Several studies have focused on short-term outcomes of in utero exposure to ADHD medications in offspring, including congenital malformations [7–15] and adverse outcomes related to labour and delivery [6, 11–13, 16–18]. However, long-term outcomes in the offspring have received less attention. A recent study from our group, conducted using Danish national registers, provided initial insights by investigating long-term neurodevelopmental and growth outcomes in children exposed to ADHD medication in utero [19]. The findings indicated no increased risk of neurodevelopmental disorders (NDDs) among exposed offspring, and this finding was later replicated in a large US study using data from publicly and commercially insured pregnant women [20]. The US study, however, included only stimulants, amphetamine/dexamfetamine and methylphenidate, whereas the Danish study also included non-stimulants (i.e., atomoxetine and clonidine), but unfortunately lacked the power to stratify the analyses by specific medication types [19]. This distinction between medication types is crucial, as the medications operate through different mechanisms of action, potentially leading to diverse effects on foetal neurodevelopment.

Currently, there are no specific guidelines for the use of any type of ADHD medication during pregnancy, largely due to insufficient evidence about their risks and benefits [21]. Only observational studies are feasible, but they come with significant limitations, especially confounding by indication, which underscores the urgent need for high-quality research. Employing triangulation approaches that combine various epidemiological designs under different assumptions can enhance the robustness of the findings. Importantly, replication studies are critical to validate previous research findings on the use of ADHD medications during pregnancy to ensure robustness of the joint evidence base. Given the increasing number of women of reproductive age using ADHD medications, there is an urgent need for evidence from multiple studies with large data sets that can inform clinical guidelines and help clinicians and patients consider if the benefits of continued ADHD medication use during pregnancy outweigh any potential teratogenic effects to the foetus [8, 22, 23]. Therefore, the present study was conducted to provide additional evidence on the association between in utero exposure to ADHD medications and offspring NDDs and to expand this knowledge by examining the associations by types of medications with data from the Swedish national registers. Importantly, this study adds to previous ones by presenting an analysis of the effects of methylphenidate, amphetamines and atomoxetine separately, in addition to examining their effects when pooled together. To increase the robustness of our results, we also performed meta-analyses of the results from the recent Danish [19] and the current Swedish study.

## Methods

### Data sources and study population

In this register-based cohort study, we drew on data from the Swedish National Registers linked via the unique personal identifier assigned to all individuals in Sweden upon birth or immigration [24]. We used the Swedish Medical Birth Register (MBR) to identify livebirths and pregnancy and birth related variables, as well as to identify mothers and fathers in relation to each included pregnancy. The MBR covers births since 1973 and has a coverage above 97% in Sweden since 2000 [25]. Medical diagnoses were identified in the National Patient Register (NPR), which contains diagnoses based on the International Classification of Diseases (ICD) codes, 10th Revision (ICD-10; 1997-onwards) from inpatient care since 1969 and outpatient specialist care since 2001 [26]. ADHD medication dispensations were identified in the Swedish Prescribed Drug Register (PDR), which contains all medications dispensed at Swedish pharmacies since 2005 coded according to the Anatomical Therapeutic Chemical (ATC) classification [27]. We used the Swedish Total Population Register to identify migrations, which includes data on all individuals living in Sweden since 1968 [24], and the Longitudinal Integration Database for Health Insurance and Labour Studies (LISA) [28] to identify sociodemographic variables, which contains data on education and labour market from Swedish inhabitants from 1990. Linked data were available until 31st December 2020.

Throughout this paper, we refer to pregnant and birthing individuals as “female,” “women,” or “mothers” for fluency. However, we acknowledge that not all individuals in our study identify with these terms.

**Fig. 1:**
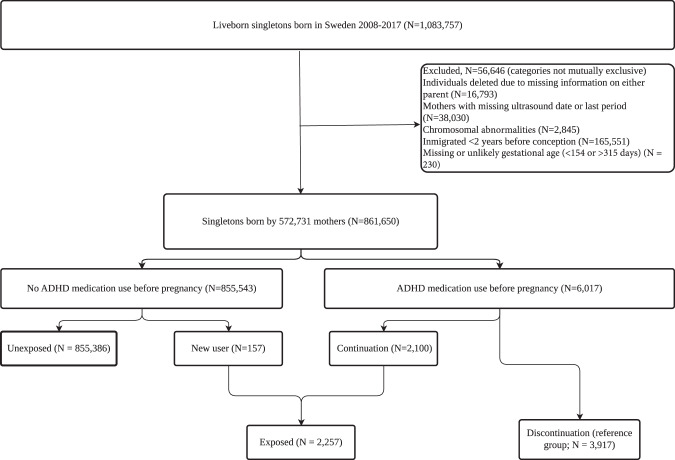
Flowchart showing identification of study population.

We identified all singletons born in Sweden between 2008 and 2017 (*N* = 1,083,757) in MBR, to allow for a two-year ascertainment period before pregnancy to identify exposure to ADHD medication and a minimum of three years follow-up for all included children to identify our outcomes (definitions below). Children with missing information on either parent, missing or unlikely gestational age (<154 or > 315 days), chromosomal abnormalities identified in the NPR [ICD-10 codes Q90–Q99), of mothers with missing data on date of ultrasound or last menstrual period, and of mothers who immigrated to Sweden < 2 years before conception were excluded. After exclusions, the final study population included 861,650 children born by 572,731 mothers (Fig. 1).

### In utero exposure to ADHD medication

In utero exposure to ADHD medication was identified from dispensation in the PDR. We considered all ADHD medications approved in Sweden during the study period, including stimulant medications (N06BA04 “methylphenidate”, N06BA01 “amphetamine”, N06BA02 “dexamfetamine”, N06BA12 “lisdexamfetamine”) and the non-stimulant medication atomoxetine (N06BA09). As in our prior work [19], ADHD medication dispensations from two years before pregnancy up to the date of delivery were included. Start of pregnancy was defined using the information on gestational age in MBR, based on the first- or second-trimester ultrasound scan or, when ultrasound data were unavailable, the first day of the mother’s last menstrual period. Exposure was categorised as follows: 1) “Unexposed” children were defined as no maternal ADHD medication dispensation in the two years prior to pregnancy up until delivery; this group consisted of the background population, but also included a smaller subset of children of mothers with an ADHD diagnosis, 2) “Discontinuation” was defined as maternal ADHD medication dispensation in the two years prior to pregnancy, not necessarily consistently, but no dispensation during pregnancy, 3) “Continuation” was defined as ADHD medication dispensation in the two years prior and during pregnancy, and 4) “New user” was defined as initiation during pregnancy or one month prior to conception but no dispensation in the two years prior. Continuation and new users were categorized as “Exposed”. We also estimated the duration of exposure to ADHD medication during pregnancy by multiplying the number of defined daily doses per package by the number of packages dispensed.

### Offspring neurodevelopmental disorders

Our main outcome was a registered diagnosis in the NPR of any neurodevelopmental disorder [ICD-codes F70–79, F84, F90–98], or, in line with prior research [5], a dispensation for any ADHD medication after the age of three years. Secondly, we also considered ADHD [F90 or prescription for ADHD medication,] and autism spectrum disorder (ASD [F84]) after age three as separate outcomes. Date of the outcome was defined by the date of the first diagnosis or the first ADHD medication dispensation, whichever came first.

### Potential confounders

We evaluated several potential confounders including: maternal age at delivery (<25, 25–34,  > 34 years), primiparity (yes/no), calendar year of delivery (2008–2011, 2012–2014, or 2015–2017) and maternal self-reported smoking during pregnancy (yes/no) identified in the MBR; Any maternal and paternal psychiatric history at delivery (ICD-8 codes 290–315, ICD-9 codes 290–319 and ICD-10 codes F00–F99) and psychiatric in- or outpatient treatment two years prior to pregnancy and until delivery (yes/no) identified in the NPR; Dispensing of other psychotropic medications during pregnancy with the ATC codes N06A antidepressants, N05A antipsychotics, N03A antiseizure, or N05B anxiolytics (yes/no) identified in the PDR; Maternal highest education (mandatory schooling to 9th grade /above mandatory school), civil status at delivery (married or cohabiting / single, divorced or widowed) identified in LISA.

### Statistical analyses

We followed each child from age 3 years until a diagnosis, death, emigration, or end of follow-up (December 31, 2020), whichever occurred first. Hazard ratios (HR) with 95% confidence intervals (CI) were estimated using Cox proportional hazards regression with the child’s attained age as the underlying time scale and cluster robust standard errors to account for the correlation between included siblings. Proportionality was evaluated by visually inspecting “log-log” plots. Children of mothers using ADHD medication during pregnancy (continuation and new users) were compared to children of mothers discontinuing ADHD medication prior to pregnancy to reduce unmeasured confounding related to maternal ADHD. We further stratified analyses in three ways. First, we stratified by medication type for methylphenidate, amphetamines (including lisdex- and dexamfetamine) and atomoxetine. Second, we stratified analyses by the timing of exposure start (first, second, and third trimester). Third, to determine if associations were modified by the duration of use, we stratified the duration of ADHD medication use during pregnancy into ≤ 90 days, 91–180 days, and ≥ 181 days. All analyses were adjusted for the maternal psychiatric and sociodemographic characteristics listed above.

Data management and statistical analyses were performed using SAS 9.4 and R version 4.3.2.

### Meta-analysis

Meta-analysis was conducted using R version 4.3.2 with the “metafor” package [29]. Hazard ratios (HR) and their corresponding 95% confidence intervals (CI) were extracted from Bang Madsen et al. [19] and current analyses. Log-transformed hazard ratios (logHR) and their standard errors (SE) were calculated to standardize the effect sizes across the two studies. Meta-analytic pooling of the hazard ratios was performed using random-effects models assuming that the true effect sizes may differ across studies. The calculation of weights was based on the inverse variance of the effect estimates. Summary estimates from the meta-analyses were exponentiated to convert logHRs back to HRs for interpretability, and results were presented with 95% CIs. Forest plots were generated to visualise the individual and pooled study estimates, along with their respective confidence intervals.

### Ethics

All methods were performed in accordance with the relevant guidelines and regulations. This study was approved by the Swedish Ethical Review Authority (reference number 2020-06540). Informed consent is not required for pseudo anonymised register-based research according to Swedish law.

### Sensitivity analyses

We performed four sensitivity analyses to address unmeasured confounding factors and misclassification of the exposure:

1. **Fathers as negative controls**: We compared children of fathers who used or discontinued ADHD medication during the index pregnancy, hypothesizing that maternal use would more directly impact intrauterine exposure. Adjustments included maternal ADHD medication use during pregnancy, paternal age at delivery, and paternal psychiatric treatment during the two years before the index pregnancy, along with variables from the main analysis.
2. **Exclusion due to co-medication**: We excluded children of mothers prescribed other psychotropic medications during pregnancy to reduce confounding by other medications or polypharmacy.
3. **Sibling design**: We used a sibling comparison design, conducting stratified Cox regression analyses on family identifiers and comparing siblings discordant in their exposure status. Adjustments included maternal use of other psychotropic medications during pregnancy, birth order, and birth year.
4. **Exclusion based on number of prescriptions**: To minimize misclassification of ADHD medication exposure, we limited analyses to mothers who filled at least two prescriptions for ADHD medication during pregnancy, ensuring higher certainty of medication consumption during pregnancy.

## Results

Among the included 861,650 liveborn children, 2257 (0.3%) were exposed to ADHD medications during pregnancy, including 2100 children whose mothers continued using ADHD medication and 157 who initiated ADHD medication during pregnancy. In total, 3917 (0.5%) children were born to mothers who discontinued ADHD medications before pregnancy; these constituted the reference group (Fig. 1). Descriptive statistics by exposure status are presented in Table 1. More mothers in the discontinuation and exposed groups had children before age 25 (44 and 35%), compared to 13% in the unexposed group, and were more likely to have children born preterm (6 and 8%) and with low birthweight (4 and 5%) compared to the unexposed group (4.5 and 2.9%). The discontinuation and exposed groups also included a larger proportion of births in more recent calendar years, first time mothers, lower education level, and smoking during pregnancy, compared to the unexposed group. The type of ADHD medications dispensed was similar between mothers in the discontinuation group and the exposed group, with the most common being methylphenidate. The only exception was lisdexamfetamine, which was more frequently used in the exposed group (10%) than in the discontinuation group (5.6%). A higher proportion of mothers in the exposed group were dispensed other psychotropic prescriptions during pregnancy (46%) and reported smoking during pregnancy (46%) compared to the discontinuation group (25 and 28%, respectively). Differences between the discontinuation and the exposed group for other psychiatric and sociodemographic variables, such as education level, civil status, and broader psychiatric history, were limited.

**Table 1:** Sociodemographic and clinical characteristics of the study population according to maternal ADHD medication use before and during pregnancy.

Characteristics	Discontinuation *n* = 3917 n (%)	Exposed *n* = 2257 n (%)	Unexposed *n* = 855,386 n (%)
**Sex of the child, female**	1913 (49)	1104 (49)	415,457 (49)
Maternal age at delivery			
<25 years	1726 (44)	781 (35)	110,958 (13)
25–34 years	1784 (46)	1162 (51)	555,465 (65)
>34 years	407 (10)	314 (14)	188,963 (22)
Calendar year of delivery			
2008–2011	629 (16)	334 (15)	360,201 (42)
2012–2014	1291 (33)	736 (33)	253,585 (30)
2015–2017	1997 (51)	1187 (53)	241,600 (28)
**Low birth weight (<2500 g)**	160 (4.1)	119 (5.3)	24,728 (2.9)
**Preterm birth (<37 weeks of gestation)**	242 (6.2)	184 (8.2)	38,198 (4.5)
**Primiparity**	2334 (60)	1176 (52)	370,094 (43)
**Any neurodevelopmental disorder**	525 (13)	284 (13)	47,140 (5.5)
ADHD	473 (12)	245 (11)	40,130 (4.7)
ASD	124 (3.2)	63 (2.8)	11,485 (1.3)
**ADHD medication type before or during pregnancy**			
Amphetamine	34 (0.9)	21 (0.9)	NA
Dexamfetamine	80 (2.0)	62 (2.7)	
Lisdexamfetamine	218 (5.6)	234 (10)	
Methylphenidate	3069 (78)	1730 (77)	
Atomoxetine	516 (13)	209 (9.3)	
**Duration of medication exposure during pregnancy**	NA		NA
<= 90 days		227 (10)	
91–180 days		158 (7.0)	
>180 days		1872 (83)	
**Timing of start of medication exposure**	NA		NA
1st trimester		1509 (67)	
2nd trimester		263 (12)	
3rd trimester		485 (21)	
**Maternal psychiatric history at delivery, yes**	3479 (89)	2085 (92)	56,143 (6.6)
**Outpatient psychiatric treatment 2 years before pregnancy to delivery, yes**	3408 (87)	2056 (91)	50,185 (5.9)
**Inpatient psychiatric treatment 2 years before pregnancy to delivery, yes**	1156 (30)	803 (36)	16,266 (1.9)
**Paternal psychiatric history at delivery, yes**	764 (20)	547 (24)	27,890 (3.3)
**Paternal outpatient psychiatric treatment 2 years before pregnancy to delivery, yes**	707 (18)	516 (23)	25,635 (3.0)
**Paternal inpatient psychiatric treatment 2 years before pregnancy to delivery, yes**	230 (5.9)	178 (7.9)	6118 (0.7)
**Maternal marital status at delivery (married or cohabiting)**	2497 (64)	1362 (60)	769,296 (90)
**Maternal highest education at delivery**			
Elementary school	1699 (43)	971 (43)	74,260 (8.7)
Above elementary school	2218 (57)	1286 (57)	781,126 (91)
**Smoking during pregnancy, yes**	1116 (28)	822 (36)	61,408 (7.2)
**Dispensing of other psychotropic prescriptions during pregnancy**			
Any	971 (25)	1037 (46)	44,963 (5.3)
Antidepressant	670 (17)	567 (25)	33,967 (4.0)
Antipsychotics	61 (1.6)	107 (4.7)	1576 (0.2)
Antiseizure	76 (1.9)	85 (3.8)	3452 (0.4)
Anxiolytics	164 (4.2)	278 (12)	5968 (0.7)

*ADHD* attention deficit hyperactivity disorder, *ASD* autism spectrum disorder.

In absolute numbers, there was no difference in the prevalence of NDDs in children of mothers in the discontinuation (13%) and exposed (13%) groups (Table 1). Mean follow-up time was 6.9 years (SD 2.3), 7.1 (2.3) and 7.2 (2.4) for NDDs overall, ADHD and ASD, respectively. Results from the Cox regressions are shown in Table 2. After adjusting for pregnancy, maternal psychiatric and sociodemographic characteristics, there was no increased hazard of any NDD (HR = 0.95, 95% 0.82;1.11), ADHD (HR = 0.92, 95% 0.78;1.08) or ASD (HR = 0.86, 95% 0.63;1.18) when comparing children in the exposed group with children in the discontinuation group. Similarly, stratified analyses by timing and duration of exposure did not show a statistically significant association between in-utero exposure to ADHD medication and any of the considered NDDs (Table 3). Stratified by medication type, results showed a consistent pattern with HRs for methylphenidate 0.94 (95% CI 0.79;1.11) and amphetamines 1.15 (95% CI 0.65;2.05) and for atomoxetine 1.03 (95% CI 0.64;1.65) for any NDD (Table 3).

**Table 2:** Results of the main analysis of the association between in utero exposure to ADHD medication and neurodevelopmental disorders in the offspring, including ADHD and ASD.

	Cases	Total number	Person years	Crude HR	Adjusted HR (95% CI)^a^
Any neurodevelopmental disorder					
ADHD medication discontinuation	525	3917	27,471	ref	ref
ADHD medication exposed	284	2257	15,660	0.97	0.95 (0.82–1.11)
ADHD					
ADHD medication discontinuation	473	3917	27,721	ref	ref
ADHD medication exposed	245	2257	15,813	0.93	0.92 (0.78–1.15)
ASD					
ADHD medication discontinuation	124	3917	28,204	ref	ref
ADHD medication exposed	63	2257	16,040	0.90	0.86 (0.63–1.18)

*ADHD* attention deficit hyperactivity disorder, *ASD* autism spectrum disorder, *HR* hazard ratio.

^a^Adjusted for maternal age at delivery, primiparity, calendar year of delivery, maternal self-reported smoking during pregnancy, any maternal and paternal psychiatric history at delivery, psychiatric in- or outpatient treatment two years prior to pregnancy and until delivery, dispensing of other psychotropic medications during pregnancy, maternal highest education, and civil status at delivery.

**Table 3:** Results of the main analysis stratified by duration of medication exposure, timing of start of medication exposure and medication types.

Stratification	Any neurodevelopmental disorder	Adjusted HR (95% CI)^a^
Duration	ADHD medication discontinuation	ref
	< = 90 days	0.88 (0.62–1.25)
	91–180 days	0.73 (0.44–1.22)
	>180 days	0.99 (0.84–1.16)
Timing of exposure	ADHD medication discontinuation	ref
	Exposure start 1st trimester	0.96 (0.81–1.14)
	Exposure start 2nd trimester	1.00 (0.71–1.40)
	Exposure start 3rd trimester	0.94 (0.71–1.26)
Medication type	ADHD medication discontinuation	ref
	Methylphenidate	0.94 (0.79–1.11)
	Amphetamines including dex- and lisdexamfetamine	1.15 (0.65–2.05)
	Atomoxetine	1.03 (0.64–1.65)
	**ADHD**	
Duration	ADHD medication discontinuation	ref
	Duration < = 90 days	0.80 (0.55–1.18)
	Duration 91–180 days	0.66 (0.38–1.17)
	Duration > 180 days	0.96 (0.81–1.15)
Timing of exposure	ADHD medication discontinuation	ref
	Exposure start 1st trimester	0.89 (0.74–1.07)
	Exposure start 2nd trimester	0.99 (0.69–1.41)
	Exposure start 3rd trimester	1.02 (0.75–1.39)
Medication type	ADHD medication discontinuation	ref
	Methylphenidate	0.89 (0.74–1.06)
	Amphetamines including dex- and lisdexamfetamine	1.05 (0.55–2.01)
	Atomoxetine	1.11 (0.68–1.82)
	**ASD**	
Duration	ADHD medication discontinuation	ref
	Duration < = 90 days	1.06 (0.54–2.07)
	Duration 91–180 days	0.75 (0.27–2.04)
	Duration > 180 days	0.84 (0.6–1.19)
Timing of exposure	ADHD medication discontinuation	Ref
	Exposure start 1st trimester	0.99 (0.7–1.39)
	Exposure start 2nd trimester	0.86 (0.4–1.84)
	Exposure start 3rd trimester	0.52 (0.26–1.03)
Medication type	ADHD medication discontinuation	ref
	Methylphenidate	0.82 (0.58–1.15)
	Amphetamines including dex- and lisdexamfetamine	1.82 (0.42–7.92)
	Atomoxetine	0.65 (0.16–2.76)

*ADHD* attention deficit hyperactivity disorder, *ASD* autism spectrum disorder, *HR* hazard ratio.

^a^Adjusted for maternal age at delivery, primiparity, calendar year of delivery, maternal self-reported smoking during pregnancy, any maternal and paternal psychiatric history at delivery, psychiatric in- or outpatient treatment two years prior to pregnancy and until delivery, dispensing of other psychotropic medications during pregnancy, maternal highest education, and civil status at delivery.

Sensitivity analyses (Table 4) confirmed the robustness of the main analyses, showing no association between ADHD medication exposure and NDDs in the offspring.

**Table 4:** Sensitivity analyses addressing different sources of biases with any neurodevelopmental disorder as the outcome.

Analysis	Potential source of bias addressed	Exposure status	Cases	Total number	Person years	Crude HR	Adjusted HR (95% CI)
Primary analysis^a^	Confounding by indication	Discontinuation	525	3917	27,471	ref	ref
		Exposed	284	2257	15,660	0.97	0.95 (0.82–1.11)
Analysis using fathers as negative control^b^	Confounding by indication	Discontinuation	264	1989	14,285	ref	ref
		Exposed	655	5010	36,834	0.91	0.97 (0.84–1.12)
Analysis excluding those with use of other psychotropic medications^c^	Confounding	Discontinuation	515	3850	27,000	ref	ref
		Exposed	274	2154	14,943	0.99	0.97 (0.83–1.13)
Sibling control analysis^d^	Confounding by family context and genetics	Non-exposed	525	3917	27,471	ref	ref
		Exposed	284	2257	15,660	0.97	1.24 (0.72–2.13)
Analysis restricting to at least two prescription fills during pregnancy^a^	Misclassification of exposure	Discontinuation	452	3362	23,609	ref	ref
		Exposed	270	2171	14,999	0.97	0.96 (0.82–1.13)

^a^Adjusted for maternal age at delivery, primiparity, calendar year of delivery, maternal self-reported smoking during pregnancy, any maternal and paternal psychiatric history at delivery, psychiatric in- or outpatient treatment two years prior to pregnancy and until delivery, dispensing of other psychotropic medications during pregnancy, maternal highest education, and civil status at delivery.

^b^Same as above including maternal ADHD medication use during pregnancy, paternal age at delivery, paternal inpatient or outpatient psychiatric treatment from two years before pregnancy to delivery.

^c^Same as in main analyses except for use of other psychotropic medications.

^d^Adjusted for maternal use of other psychotropic medications during pregnancy, birth order, and birth year.

**Fig. 2:**
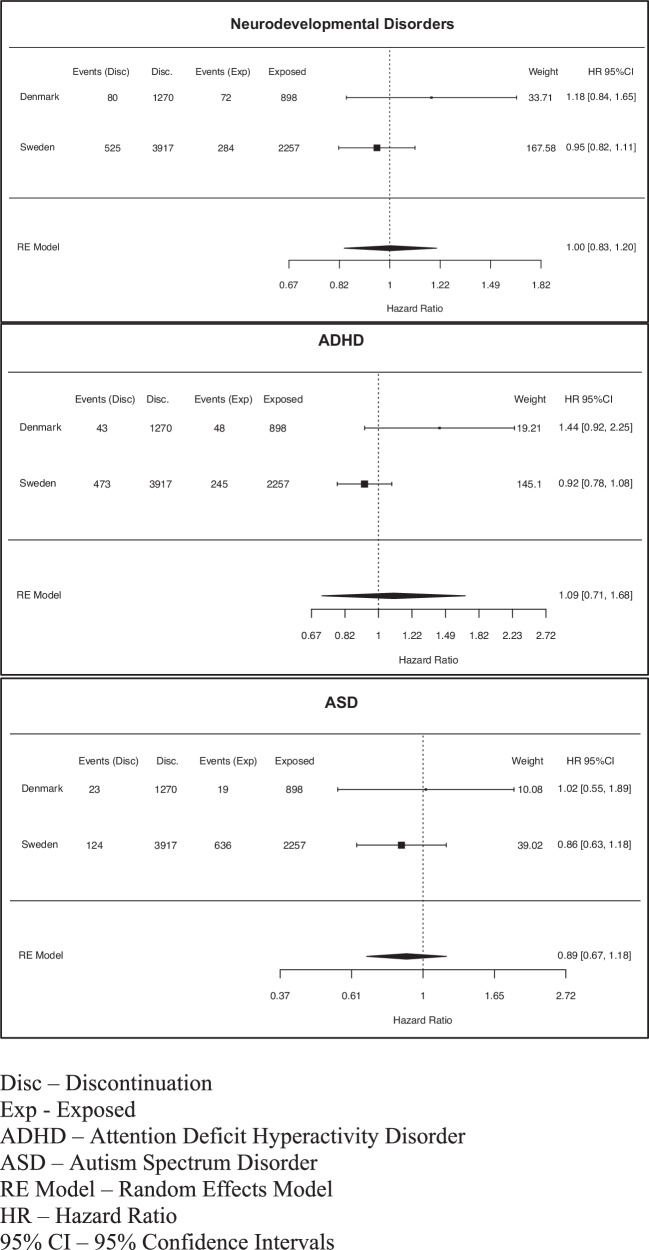
Forest plots of the meta-analyses of the Danish and Swedish data.

Forest plots of the meta-analyses are shown in Fig. 2. With a total population of 3155 children exposed and 5187 in the discontinuation group, pooled HR estimates for any NDD, ADHD, and ASD were 1.00 (95% CI 0.83;1.20), 1.09 (95% CI 0.71;1.68), and 0.89 (95% CI 0.67;1.18), respectively.

## Discussion

In this large Swedish population-based cohort study, we replicate the previous findings of no association between in utero exposure to methylphenidate and amphetamines and risk of neurodevelopmental disorders in the offspring and extend the evidence by providing separate analyses for the non-stimulant ADHD medication atomoxetine, showing no increased risk either. Analyses stratified by timing and duration of exposure also showed no significant associations. Sensitivity analyses further adjusting for familial confounding through sibling analyses and using fathers as negative controls also confirmed these findings.

Our study leverages the comprehensive data available in Swedish national registers to replicate and extend the findings from previous studies [19, 20, 30], providing an important test of the initial conclusions that in utero exposure to ADHD medication does not increase the risk of NDDs in the offspring. Replication of findings in different contexts is crucial for validating the generalizability of research outcomes. Differences in healthcare systems, medication use patterns, and patient demographics can all influence study results. While there are similarities between the healthcare systems of Denmark and Sweden, several differences might influence the population of women who use ADHD medication during pregnancy. One notable difference is the accessibility and prevalence of ADHD medication use. In Sweden, the use of ADHD medication, both during pregnancy and in general, is significantly more common compared to Denmark [4, 31]. Also, in Sweden, the number of psychiatrists per capita is much higher [32] and citizens can self-refer for psychiatric assessment, bypassing primary care, whereas in Denmark, a referral from a primary care provider is required. This difference in healthcare pathways could lead to variations in the demographics and characteristics of women who use ADHD medication during pregnancy in these two countries, thereby creating a different confounder structure. Reassuringly, there are no indications that underlying variation in ADHD incidence and variability related to structural differences in the organization of health care seems to influence the results.

Through this rigorous scientific approach, we contribute to a clearer understanding of the safety of ADHD medication use during pregnancy, aiding healthcare providers and patients in making informed decisions about ADHD medication management in pregnancy. Our study also extends the findings from previous studies by providing results on the non-stimulant medication atomoxetine, showing no evidence of an increased risk of NDDs even for this compound.

As current guidelines do not include specific indications on the use of ADHD medication in pregnancy, our results are relevant as they can inform future guidelines on the treatment of ADHD.

Future research should focus on further elucidating the long-term safety of ADHD medications during pregnancy, including potential effects on other developmental domains not covered in this study. Studies with larger sample sizes and more detailed information on dosage and adherence could provide more granular insights. Additionally, research exploring the underlying mechanisms through which ADHD medications may or may not influence foetal development would be valuable.

Although our primary analysis focused on the risk of neurodevelopmental disorders in children exposed to ADHD medication in utero, descriptive analyses revealed a higher cumulative incidence of preterm birth and low birth weight among the exposed group. However, as these comparisons were not adjusted for potential confounders, the observed differences should be interpreted with caution. It is unclear whether these outcomes reflect a direct effect of ADHD medication exposure or are driven by unmeasured confounders, such as maternal lifestyle factors (e.g., smoking, diet, and stress), co-existing psychiatric conditions, or broader health challenges.

### Strengths and limitations

The main strength of our study lies in the use of comprehensive, high-quality data from the Swedish national registers, allowing for a detailed and robust analysis of a large cohort. The extensive follow-up period and the ability to control for a wide range of confounding factors, including maternal psychiatric history and sociodemographic characteristics, further strengthen our finding. An important strength is the analysis of the effects of stimulants and non-stimulants separately, which is informative for shared decision-making in clinical practice. Additionally, the replication of previous studies’ results and the inclusion of meta-analyses enhance the reliability and generalizability of our conclusions.

Limitations must also be considered. Despite the nationwide nature of the study data and large sample size, statistical analyses were underpowered as indicated by the wide confidence intervals. Further, despite rigorous adjustments, residual confounding cannot be entirely ruled out, particularly regarding unmeasured genetic and environmental factors. Although the registers capture a range of important covariates, they do not include information on important factors related to maternal lifestyle during pregnancy such as alcohol and illegal drug use during pregnancy. However, diagnosed alcohol abuse was accounted for as a covariate if the woman had an in- or outpatient hospital contact related to substance use disorder during the two years before pregnancy up to delivery. Whilst we used sibling comparison to partly address this, this only accounts for ~50% of segregating genes and environmental factors that are shared by siblings.

As with other pharmacoepidemiologic studies using register data, there is a risk of misclassification when defining exposure status based on redeemed prescriptions [33]. To address this, we conducted sensitivity analyses by (1) restricting the exposed group to those who redeemed at least two prescriptions during pregnancy and (2) stratifying by duration of ADHD medication exposure, with the longest duration being more than 180 days. Our findings remained robust across these analyses. However, the lack of clinical information about the doses of ADHD medication taken could have led to an underestimation of exposure time, as pregnant women might lower their dose during pregnancy or physicians might prescribe a lower dose.

## Conclusions

Our study supports the safety of using ADHD medication during pregnancy in relation to neurodevelopmental disorders (NDDs) in offspring. These findings contribute to the growing evidence base needed to inform clinical guidelines and ensure that treatment decisions during pregnancy are based on robust and reliable data.

For new users, methylphenidate appears to be the ‘safest’ choice, given that it is the most thoroughly investigated ADHD medication. However, for patients who are already doing well on amphetamines or atomoxetine, there is currently no compelling reason to switch medications based on safety concerns regarding long-term outcomes. It is important to note that the sample sizes for these groups are relatively small, and more data on these medication classes are needed to confirm these findings.

Overall, our study provides reassuring evidence that continuing ADHD medication during pregnancy does not increase the risk of long-term NDDs in offspring. This information is crucial for healthcare providers and expectant mothers when making informed decisions about ADHD medication management during pregnancy. Future research should continue to explore the long-term safety of various ADHD medications during pregnancy, including further investigation into specific medication classes and their potential effects on other developmental domains.

## Data Availability

Access to individual-level data from Sweden is governed by Swedish authorities. Each scientific project must be approved before initiation, and approval is granted to a specific Swedish research institution. Researchers at Swedish research institutions may obtain the relevant approval and data. International researchers may gain data access if governed by a Swedish research institution having needed approval and data access.

## Code availability

Code can be made accessible upon reasonable request.

## References

[1] Faraone SV, Bellgrove MA, Brikell I, Cortese S, Hartman CA, Hollis C, et al. Attention-deficit/hyperactivity disorder. Nat Rev Dis Primers. 2024;10:11. 38388701 10.1038/s41572-024-00495-0

[2] Anderson KN, Ailes EC, Danielson M, Lind JN, Farr SL, Broussard CS, et al. Attention-deficit/Hyperactivity disorder medication prescription claims among privately insured women aged 15–44 years - United States, 2003–2015. MMWR Morb Mortal Wkly Rep. 2018;67:66–70. 29346342 10.15585/mmwr.mm6702a3 PMC5772805

[3] Louik C, Kerr S, Kelley KE, Mitchell AA. Increasing use of ADHD medications in pregnancy. Pharmacoepidemiol Drug Saf. 2015;24:218–20. 25630904 10.1002/pds.3742 PMC4313616

[4] Cohen JM, Srinivas C, Furu K, Cesta CE, Reutfors J, Karlstad Ø. Prevalence trends and individual patterns of ADHD medication use in pregnancy in Norway and Sweden, 2010–2019. Eur J Clin Pharmacol. 2023;79:173–80. 36445458 10.1007/s00228-022-03428-6 PMC9816174

[5] Bang Madsen K, Bliddal M, Skoglund CB, Larsson H, Munk-Olsen T, Madsen MG, et al. Attention-deficit hyperactivity disorder (ADHD) medication use trajectories among women in the perinatal period. CNS Drugs. 2024;38:303–14. 38489019 10.1007/s40263-024-01076-1 PMC10980654

[6] Li L, Sujan AC, Butwicka A, Chang Z, Cortese S, Quinn P, et al. Associations of prescribed ADHD medication in pregnancy with pregnancy-related and offspring outcomes: a systematic review. CNS Drugs. 2020;34:731–47. 32333292 10.1007/s40263-020-00728-2 PMC7338246

[7] Haervig KB, Mortensen LH, Hansen AV, Strandberg-Larsen K. Use of ADHD medication during pregnancy from 1999 to 2010: a Danish register-based study. Pharmacoepidemiol Drug Saf. 2014;23:526–33. 24590619 10.1002/pds.3600

[8] Damer EA, Edens MA, van der Loos MLM, van Esenkbrink J, Bunkers I, van Roon EN, et al. Fifteen years’ experience with methylphenidate for attention-deficit disorder during pregnancy: effects on birth weight, apgar score and congenital malformation rates. Gen Hosp Psychiatry. 2021;73:9–15. 34507078 10.1016/j.genhosppsych.2021.09.003

[9] Damkier P, Broe A. First-trimester pregnancy exposure to modafinil and risk of congenital malformations. JAMA. 2020;323:374–6. 31990303 10.1001/jama.2019.20008 PMC6990936

[10] Huybrechts KF, Bröms G, Christensen LB, Einarsdóttir K, Engeland A, Furu K, et al. Association between methylphenidate and amphetamine use in pregnancy and risk of congenital malformations: A Cohort Study From the International Pregnancy Safety Study Consortium. JAMA Psychiatry. 2018;75:167–75. 29238795 10.1001/jamapsychiatry.2017.3644 PMC5838573

[11] Nörby U, Winbladh B, Källén K. Perinatal outcomes after treatment with ADHD medication during pregnancy. Pediatrics. 2017;140:e20170747. 29127207 10.1542/peds.2017-0747

[12] Diav-Citrin O, Shechtman S, Arnon J, Wajnberg R, Borisch C, Beck E, et al. Methylphenidate in pregnancy: a multicenter, prospective, comparative, observational study. J Clin Psychiatry. 2016;77:1176–81. 27232650 10.4088/JCP.15m10083

[13] Bro SP, Kjaersgaard MI, Parner ET, Sørensen MJ, Olsen J, Bech BH, et al. Adverse pregnancy outcomes after exposure to methylphenidate or atomoxetine during pregnancy. Clin Epidemiol. 2015;7:139–47. 25657597 10.2147/CLEP.S72906 PMC4317061

[14] Pottegård A, Hallas J, Andersen JT, Løkkegaard EC, Dideriksen D, Aagaard L, et al. First-trimester exposure to methylphenidate: a population-based cohort study. J Clin Psychiatry. 2014;75:e88–93. 24502866 10.4088/JCP.13m08708

[15] Anderson KN, Dutton AC, Broussard CS, Farr SL, Lind JN, Visser SN, et al. ADHD Medication use during pregnancy and risk for selected birth defects: National Birth Defects Prevention Study, 1998–2011. J Atten Disord. 2020;24:479–89. 29519207 10.1177/1087054718759753 PMC6119527

[16] Poulton AS, Armstrong B, Nanan RK. Perinatal outcomes of women diagnosed with attention-deficit/hyperactivity disorder: An Australian Population-Based Cohort Study. CNS Drugs. 2018;32:377–86. 29557079 10.1007/s40263-018-0505-9

[17] Cohen JM, Hernández-Díaz S, Bateman BT, Park Y, Desai RJ, Gray KJ, et al. Placental complications associated with psychostimulant use in pregnancy. Obstet Gynecol. 2017;130:1192–201. 29112657 10.1097/AOG.0000000000002362 PMC5709205

[18] Jiang H-Y, Zhang X, Jiang C-M, Fu H-B. Maternal and neonatal outcomes after exposure to ADHD medication during pregnancy: a systematic review and meta-analysis. Pharmacoepidemiol Drug Saf. 2019;28:288–95. 30585374 10.1002/pds.4716

[19] Bang Madsen K, Robakis TK, Liu X, Momen N, Larsson H, Dreier JW, et al. In utero exposure to ADHD medication and long-term offspring outcomes. Mol Psychiatry. 2023;28:1739–46. 36759544 10.1038/s41380-023-01992-6

[20] Suarez EA, Bateman BT, Hernandez-Diaz S, Straub L, McDougle CJ, Wisner KL, et al. Prescription stimulant use during pregnancy and risk of neurodevelopmental disorders in children. JAMA Psychiatry. 2024;81:477–88. 38265792 10.1001/jamapsychiatry.2023.5073 PMC10809143

[21] McAllister-Williams RH, Baldwin DS, Cantwell R, Easter A, Gilvarry E, Glover V, et al. British association for psychopharmacology consensus guidance on the use of psychotropic medication preconception, in pregnancy and postpartum 2017. J Psychopharmacol. 2017;31:519–52. 28440103 10.1177/0269881117699361

[22] Chang Z, Ghirardi L, Quinn PD, Asherson P, D’Onofrio BM, Larsson H. Risks and benefits of attention-deficit/hyperactivity disorder medication on behavioral and neuropsychiatric outcomes: a qualitative review of pharmacoepidemiology studies using linked prescription databases. Biol Psychiatry. 2019;86:335–43. 31155139 10.1016/j.biopsych.2019.04.009 PMC6697582

[23] Russell DJ, Wyrwoll CS, Preen DB, Kelty E. Investigating maternal and neonatal health outcomes associated with continuing or ceasing dexamphetamine treatment for women with attention-deficit hyperactivity disorder during pregnancy: a retrospective cohort study. Arch Womens Ment Health. 2024;27:785–94. 38424254 10.1007/s00737-024-01450-4 PMC11405422

[24] Ludvigsson JF, Almqvist C, Bonamy AE, Ljung R, Michaelsson K, Neovius M, et al. Registers of the Swedish total population and their use in medical research. Eur J Epidemiol. 2016;31:125–36. 26769609 10.1007/s10654-016-0117-y

[25] Cnattingius S, Källén K, Sandström A, Rydberg H, Månsson H, Stephansson O, et al. The Swedish medical birth register during five decades: documentation of the content and quality of the register. Eur J Epidemiol. 2023;38:109–20. 36595114 10.1007/s10654-022-00947-5 PMC9867659

[26] Ludvigsson JF, Andersson E, Ekbom A, Feychting M, Kim JL, Reuterwall C, et al. External review and validation of the Swedish national inpatient register. BMC Public Health. 2011;11:450. 21658213 10.1186/1471-2458-11-450 PMC3142234

[27] Wettermark B, Hammar N, Fored CM, Leimanis A, Otterblad Olausson P, Bergman U, et al. The new Swedish prescribed drug register–opportunities for pharmacoepidemiological research and experience from the first six months. Pharmacoepidemiol Drug Saf. 2007;16:726–35. 16897791 10.1002/pds.1294

[28] Ludvigsson JF, Svedberg P, Olén O, Bruze G, Neovius M. The longitudinal integrated database for health insurance and labour market studies (LISA) and its use in medical research. Eur J Epidemiol. 2019;34:423–37. 30929112 10.1007/s10654-019-00511-8 PMC6451717

[29] Viechtbauer W. Conducting meta-analyses in R with the metafor package. J Stat Softw. 2010;36:1–48.

[30] Lemelin M, Sheehy O, Zhao JP, Bérard A. Maternal ADHD medication use during pregnancy and the risk of ADHD in children: importance of genetic predispositions and impact of using a sibling analysis. Eur Neuropsychopharmacol. 2021;44:66–78. 33461830 10.1016/j.euroneuro.2021.01.003

[31] Sørensen AMS, Wesselhöeft R, Andersen JH, Reutfors J, Cesta CE, Furu K, et al. Trends in use of attention deficit hyperactivity disorder medication among children and adolescents in Scandinavia in 2010–2020. Eur Child Adolesc Psychiatry. 2023;32:2049–56. 35831669 10.1007/s00787-022-02034-2

[32] Barrett E, Jacobs B, Klasen H, Herguner S, Agnafors S, Banjac V, et al. The child and adolescent psychiatry: study of training in Europe (CAP-STATE). Eur Child Adolesc Psychiatry. 2020;29:11–27. 31845068 10.1007/s00787-019-01416-3

[33] Rasmussen L, Wettermark B, Steinke D, Pottegård A. Core concepts in pharmacoepidemiology: measures of drug utilization based on individual-level drug dispensing data. Pharmacoepidemiol Drug Saf. 2022;31:1015–26. 35819240 10.1002/pds.5490 PMC9545237

